# Supplementary material for: Multifunctional Chitosan/Gold Nanoparticles Coatings for Biomedical Textiles
Source: Nanomaterials (Basel). 2019 Jul 24;9(8):1064. doi: 10.3390/nano9081064 (PMC6723569; doi:10.3390/nano9081064)
Supplement: Supplementary file 1 [file nanomaterials-09-01064-s001.pdf]

Supplementary Material

Multifunctional Chitosan/Gold Nanoparticles Coatings for Biomedical Textiles

Iris O. Silva[1], Rasiah Ladchumananandasivam[1], José Heriberto O. Nascimento[2], Késia Karina O.S. Silva[2], Fernando R. Oliveira[3], António Pedro Souto[4], Helena P. Felgueiras[4], and Andrea Zille[4*]

[1]Department of Mechanical Engineering, Federal University of Rio Grande do Norte, Natal, Brazil

[2]Department of Textile Engineering, Federal University of Rio Grande do Norte, Natal, Brazil

[3]Department of Textile Engineering, Federal University of Santa Catarina (UFSC), Campus Blumenau, Brazil

[4]Centre for Textile Science and Technology, Department of Textile Engineering, University of Minho, Guimarães, Portugal

***Corresponding Author:** Andrea Zille, E-mail: azille@2c2t.uminho.pt

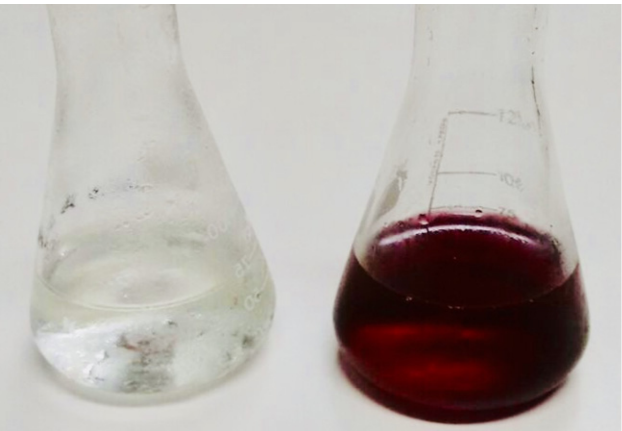

Figure S1. Solution of $HAuCl_4 \cdot 3H_2O$ (0.25 mM) before and after citrate reduction for AuNPs production.

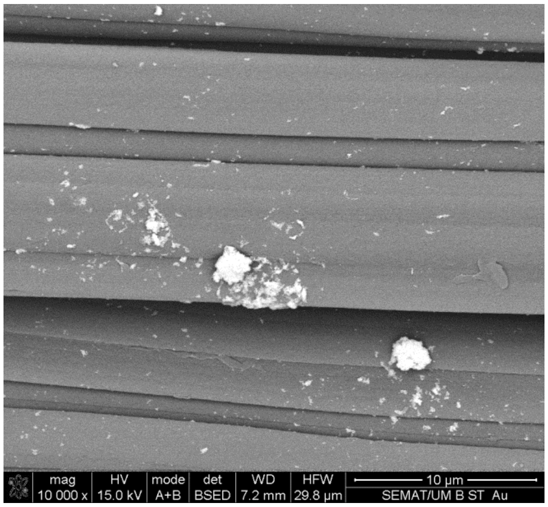

Figure S2. SEM micrograph of AuNPs functionalized soybean knitted fabrics without chitosan pre-treatment, captured at 10,000x magnification. Evidences of formation of AuNPs aggregates.

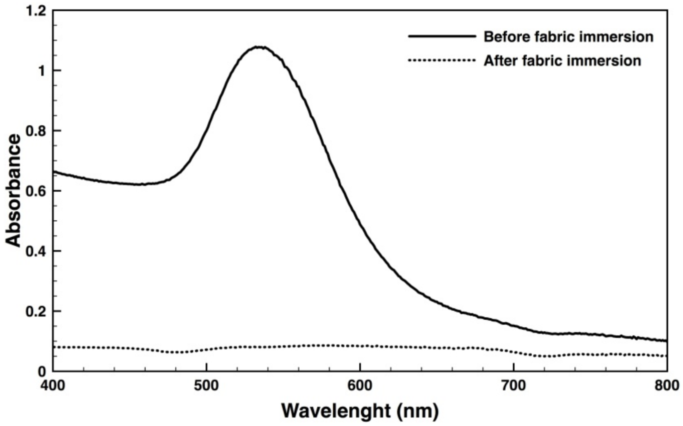

Figure S3. UV-visible spectra of AuNPs colloidal suspension (400-800 nm), before and after chitosan-treated soybean fabric immersion.

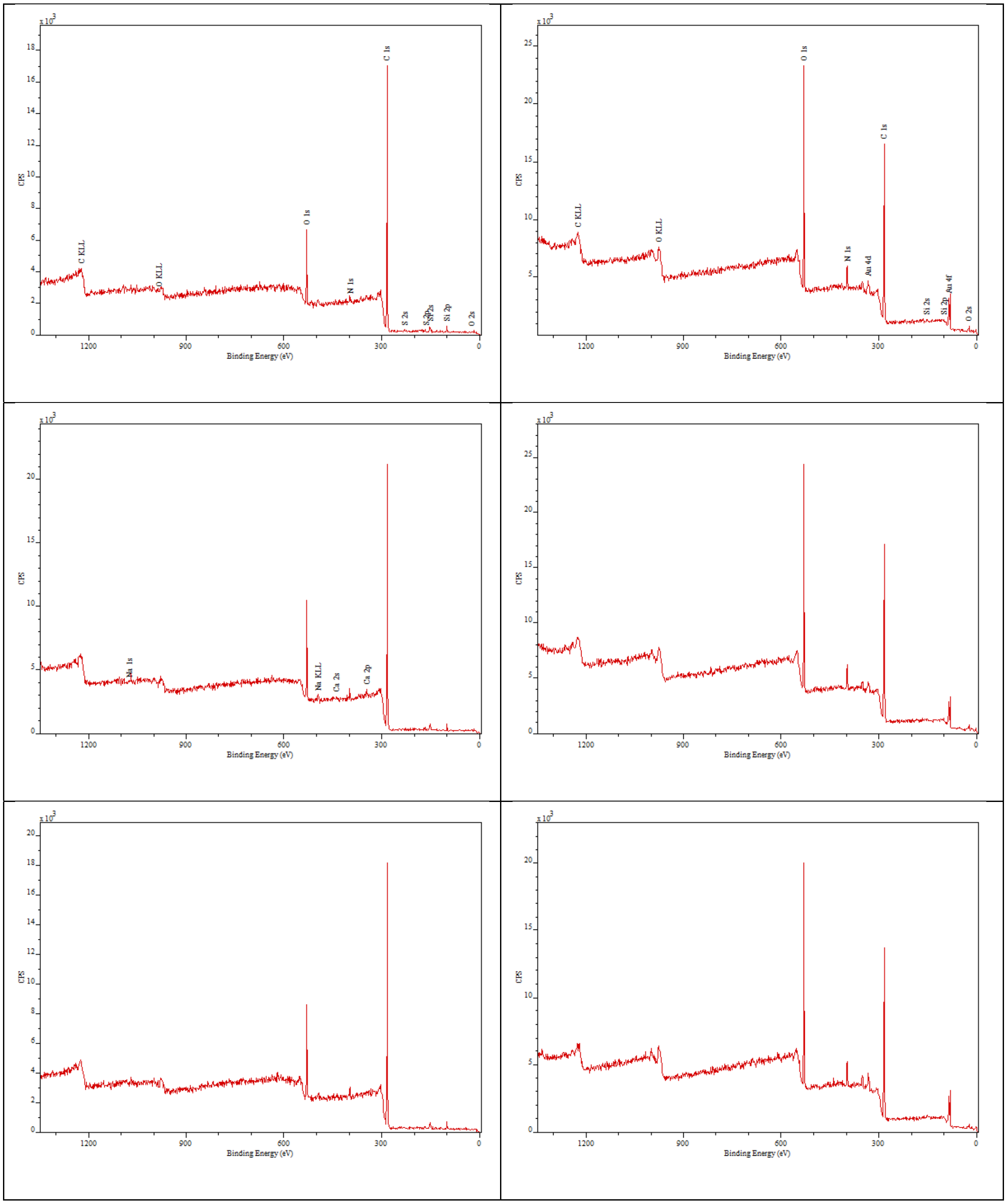

Figure S4. Survey XPS spectra of the Soybean fibers surface control (left column) and with deposited Chitosan+AuNPs (right column).

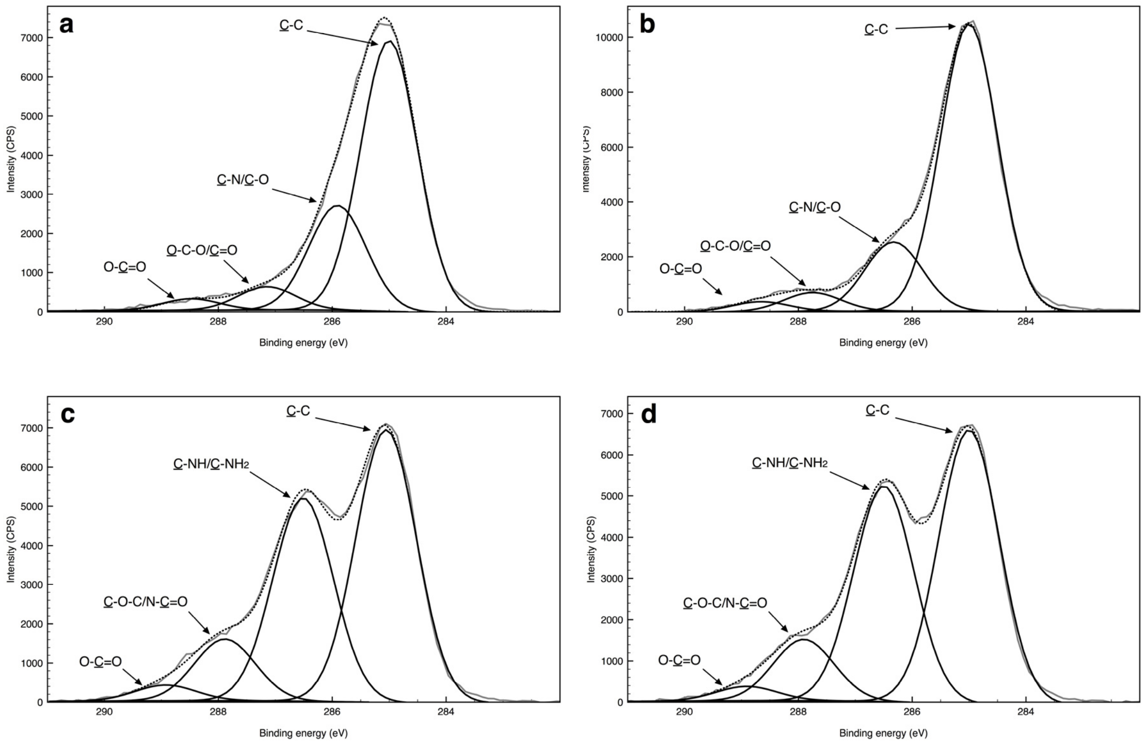

Figure S5 - High-resolution deconvoluted XPS spectra of the C1s binding energy region of Soybean fibers surface (a), with deposited AuNPs (b), after chitosan treatment (c) and after AuNPs deposition in chitosan treated fibers (d).

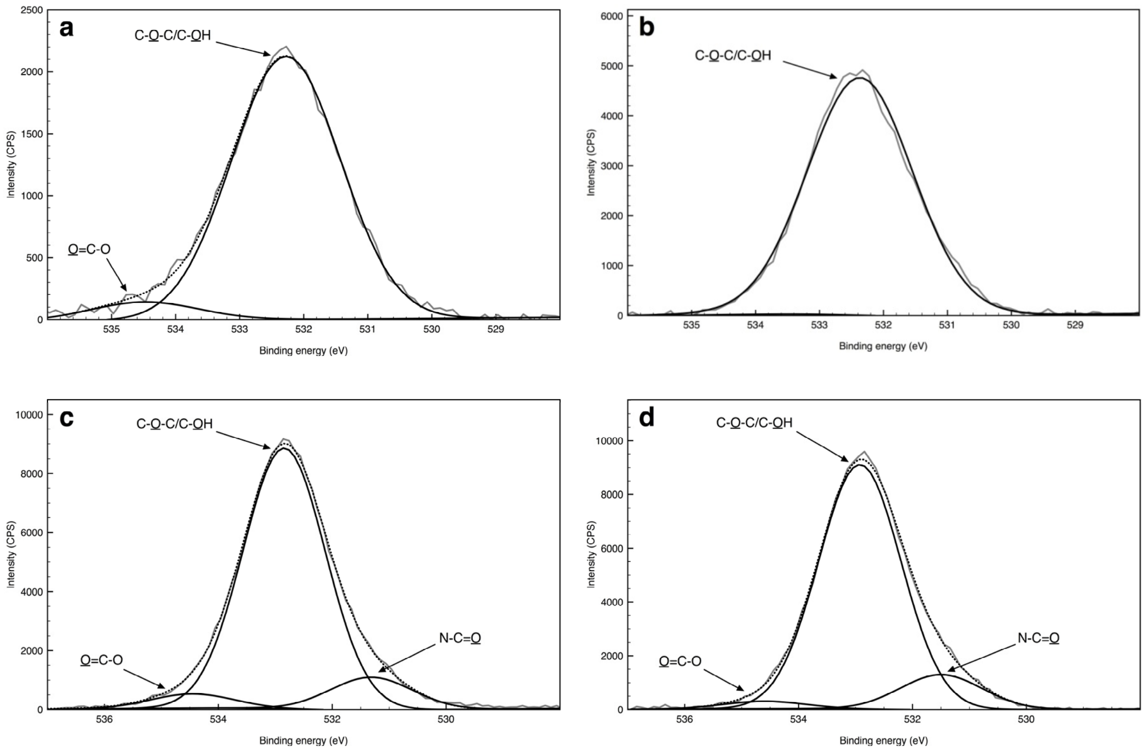

Figure S6 - High-resolution deconvoluted XPS spectra of the O1s binding energy region of Soybean fibers surface (a), with deposited AuNPs (b), after chitosan treatment (c) and after AuNPs deposition in chitosan treated fibers (d).

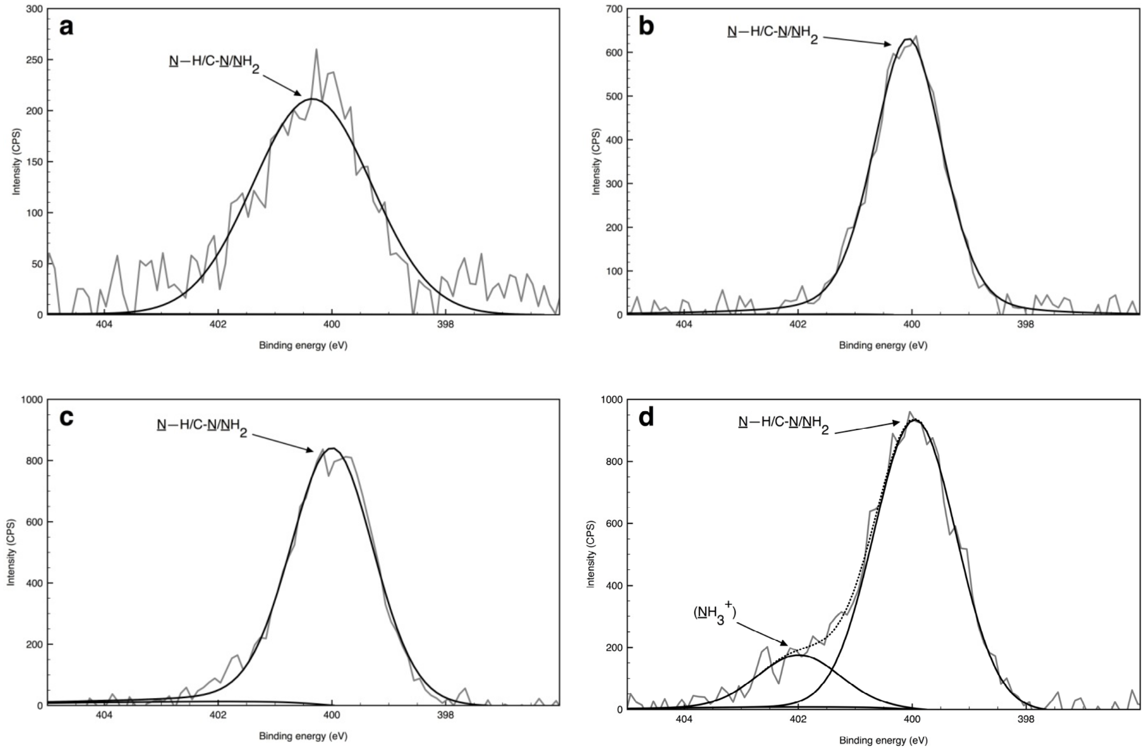

Figure S7 - High-resolution deconvoluted XPS spectra of the N1s binding energy region of Soybean fibers surface (a), with deposited AuNPs (b), after chitosan treatment (c) and after AuNPs deposition in chitosan treated fibers (d).

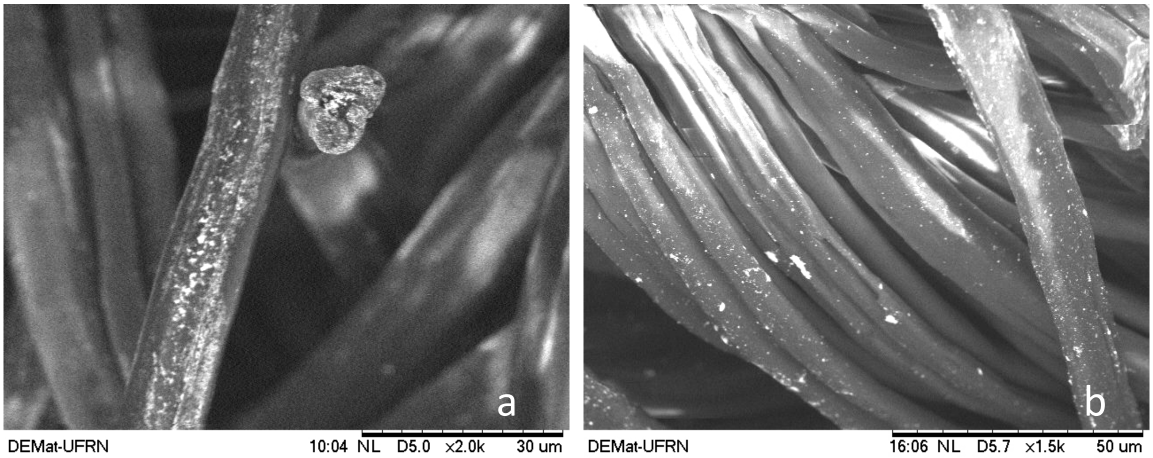

Figure S8 - SEM micrographs at different magnifications of chitosan prep-treated soybean fabrics with deposited AuNPs (a) before and (b) after 5 washing cycles.
